# Identification of a stemness-related gene panel associated with BET inhibition in triple negative breast cancer

**DOI:** 10.1007/s13402-020-00497-6

**Published:** 2020-03-12

**Authors:** Leticia Serrano-Oviedo, Miriam Nuncia-Cantarero, Sara Morcillo-Garcia, Cristina Nieto-Jimenez, Miguel Burgos, Veronica Corrales-Sanchez, Javier Perez-Peña, Balázs Győrffy, Alberto Ocaña, Eva María Galán-Moya

**Affiliations:** 1 Translational Oncology Laboratory, Centro Regional de Investigaciones Biomédicas (CRIB), Universidad de Castilla-La Mancha: Campus de Albacete, C/ Almansa 14, 02008 Albacete, Spain; 2 Translational Research Unit, CIBERONC and Complejo Hospitalario Universitario de Albacete, Albacete, Spain; 3 Physiology and Cell Dynamics Laboratory, Centro Regional de Investigaciones Biomédicas (CRIB), Universidad de Castilla-La Mancha: Campus de Albacete, Albacete, Spain; 4 Semmelweis University 2nd Dept. of Pediatrics, and MTA TTK Lendület Cancer Biomarker Research Group, Institute of Enzymology, Hungarian Academy of Sciences, Budapest, Hungary; 5 Experimental Therapeutics Unit, Medical Oncology Department, Hospital Clínico San Carlos and IdISSC, Madrid, Spain

**Keywords:** Triple negative breast cancer, BET inhibitors, Biomarkers, Targeted therapy, Cancer stem cells, Tumour heterogeneity, Stemness

## Abstract

**Purpose:**

Triple negative breast cancers (TNBCs) are enriched in cells bearing stem-like features, i.e., cancer stem cells (CSCs), which underlie cancer progression. Thus, targeting stemness may be an interesting treatment approach. The epigenetic machinery is crucial for maintaining the stemness phenotype. Bromodomain and extra-terminal domain (BET) epigenetic reader family members are emerging as novel targets for cancer therapy, and have already shown preclinical effects in breast cancer. Here, we aimed to evaluate the effect of the BET inhibitor JQ1 on stemness in TNBC.

**Methods:**

Transcriptomic, functional annotation and qRT-PCR studies were performed on JQ1-exposed TNBC cells in culture. The results obtained were confirmed in spheroids and spheroid-derived tumours. In addition, limiting dilution, secondary and tertiary tumour sphere formation, matrigel invasion, immunofluorescence and flow cytometry assays were performed to evaluate the effect of JQ1 on CSC features. For clinical outcome analyses, the online tool Kaplan-Meier Plotter and an integrated response database were used.

**Results:**

We found that JQ1 modified the expression of stemness-related genes in two TNBC-derived cell lines, MDA-MB-231 and BT549. Among these changes, the *CD44* Antigen/*CD24* Antigen (*CD44/CD24*) ratio and Aldehyde Dehydrogenase 1 Family Member A1 (*ALDH1A1*) expression level, i.e., both classical stemness markers, were found to be decreased by JQ1. Using a validated spheroid model to mimic the intrinsic characteristics of CSCs, we found that JQ1 decreased surface CD44 expression, inhibited self-renewal and invasion, and induced cell cycle arrest in G0/G1, thereby altering the stemness phenotype. We also found associations between four of the identified stemness genes, Gap Junction Protein Alpha 1 (*GJA1*)*, CD24,* Epithelial Adhesion Molecule (*EPCAM*) and SRY-related HMG-box gene 9 (*SOX9*), and a worse TNBC patient outcome. The expression of another two of the stemness-related genes was found to be decreased by JQ1, i.e., ATP Binding Cassette Subfamily G Member 2 (*ABCG2*) and *RUNX2*, and predicted a low response to chemotherapy in TNBC patients, which supports a role for *RUNX2* as a potential predictive marker for chemotherapy response in TNBC.

**Conclusions:**

We identified a stemness-related gene panel associated with JQ1 and describe how this inhibitor modifies the stemness landscape in TNBC. Therefore, we propose a novel role for JQ1 as a stemness-targeting drug. Loss of the stem cell phenotype via JQ1 treatment could lead to less aggressive and more chemo-sensitive tumours, reflecting a better patient prognosis. Thus, the identified gene panel may be of interest for the clinical management of patients with aggressive TNBC.

**Electronic supplementary material:**

The online version of this article (10.1007/s13402-020-00497-6) contains supplementary material, which is available to authorized users.

## Introduction

Cancer progression involves loss of differentiation and acquisition of multipotency and asymmetric division characteristics [1]. Accordingly, some cancer cells adopt stem cell-like features, such as self-renewal and the ability to differentiate into multiple cell lineages from the tissue of origin [2]. This subpopulation of cells also shows an increased expression of stem cell-related genes [3] and, as such, they have been dubbed cancer stem cells (CSCs). CSCs participate in a number of processes during cancer progression, i.e., they are intrinsically resistant to standard chemotherapies, play a central role in cancer metastasis and appear to be responsible for tumour recurrence [4–6]. Together, these characteristics are known as cancer stemness [7].

Also, abnormal epigenetic gene regulation may contribute to cancer initiation and progression, and affect the stemness phenotype [8, 9]. Through a deregulated epigenetic program, tumours can increase the number of CSCs, suggesting that the epigenetic machinery is essential for the maintenance of stem cell identity. Consequently, targeting epigenetic-related mechanisms may be a promising strategy to inhibit this cancer cell sub-population [10]. Several epigenetic inhibitors are currently under clinical evaluation for cancer treatment, among which inhibitors of histone deacetylases (HDACs), enhancer of Zeste homolog 2 (EZH2), DNA methyltransferases (DNMTs), lysine-specific demethylase 1 (LSD1), and bromodomain and extra-terminal domain (BET) family proteins [11, 12]. The BET family of proteins, including BRD2, BRD3, BRD4 and BRDT, plays a crucial role in epigenetic regulation of gene transcription through the ability to recognise histone acetylated lysine residues. These epigenetic readers have recently emerged as new therapeutic targets for cancer, as well as for metabolic and inflammatory diseases [8, 13, 14]. These epigenetic drugs exhibit antiproliferative effects through a complex mechanism that involves gene transcription inhibition, cell cycle arrest and, therefore, altered cell division. In addition, BET inhibitors (BETi) have shown preclinical activity in breast cancer [13–15], although, the use of BETi as single agents in clinical trials has so far yielded modest results [15].

Triple negative breast cancer (TNBC) is a subtype of breast cancer for which currently limited therapeutic options exist. This is because it lacks specific targets that can be used to guide therapy. Although aggressive chemotherapy can lead to an initial high response rate, TNBC patients often develop resistance to chemotherapy, and distant relapses are frequently observed [16–18]. Consequently, effective treatment of TNBC remains an unmet need and the identification of targets to treat this subtype is essential for improved therapeutic efficacy. Resistance to chemotherapy usually occurs in CSCs, which exhibit a low proliferation rate [5]. Similarly, tumour relapses are associated with the presence of dormant CSCs [19]. As such CSCs are, at least partly, considered to be responsible for the high degree of therapy resistance in TNBC [20]. Therefore, targeting stemness may be a potential strategy to address this disease.

Here, through transcriptomic and functional annotation analyses, we identified a panel of stemness-related genes that can efficiently be targeted with the BETi JQ1. Moreover, we found that JQ1 impaired several CSC features in a three-dimensional (3D) model of TNBC, including growth under non-adherent conditions, self-renewal and expression of stemness marker genes. Importantly, we found that JQ1-mediated downregulation of this stemness signature was linked to improved TNBC patient prognosis, and that two of the genes identified, *ABCG2* and *RUNX2*, have the ability to predict response to chemotherapy.

## Material and methods

### Cell culture and drug compounds

MDA-MB-231 and BT-549 TNBC cells were grown as adherent monolayers in Dulbecco’s Modified Eagle’s Medium (DMEM) containing 10% Foetal Bovine Serum (FBS), 100 U/ml penicillin, 100 μg/ml streptomycin and 2 mM L-glutamine (all from Sigma Aldrich, Merck KGaA, Darmstadt, Germany), in a 5% CO_2_ atmosphere at 37 °C.

Spheroid models were designed as follows: a monolayer of adherent MDA-MB-231 or BT-549 cells was extensively washed with Phosphate-Buffered Saline (PBS) and gently scraped to detach them from the plastic surface. Next, the cells were centrifuged at 900 rpm for 5 min and transferred to non-adherent plates containing a CSC-defined medium (FBS-free Dulbecco’s Modified Eagle’s Medium Nutrient Mixture F-12 Ham, DMEM F-12) (Sigma Aldrich, Merck KGaA, Darmstadt, Germany) containing 100 U/ml penicillin, 100 μg/ml streptomycin, B27 supplement (Invitrogen, Thermo Fisher Scientific Inc.), EFG (20 ng/ml) and FGF (20 ng/ml) (Sigma Aldrich, Merck KGaA, Darmstadt, Germany) for at least 21 days to generate primary spheroids. The BETi JQ1 and OTX-015 were purchased from Selleckchem company.

### Transcriptomic, functional enrichment and gene expression analyses

MDA-MB-231 cells were exposed to JQ1 (500 nM) for 12 and 24 h. Next, RNA was isolated using a Qiagen RNeasy kit following the manufacturer’s instructions. DNase treatment was performed on the RNA samples. Transcriptomic analyses were performed using an Affymetrix Human Transcriptome Array 2.0 at the Genomic Platform of the Cancer Institute of Salamanca. To identify cellular functions altered by JQ1 treatment, the differentially expressed genes were analysed using DAVID Bioinformatics Resources 6.7 (https://david.ncifcrf.gov/summary.jsp), a tool to perform gene set enrichment analyses. An adjusted *p* value < 0.05 was used to select the five highest ranked enriched gene-sets. Next, deregulated genes were classified according to their involvement in a wide range of cellular functions using Gene Set Enrichment Analysis (GSEA) software available at http://software.broadinstitute.org/gsea/index.jsp. Among the potentially affected functions, we searched for those related to cancer stemness. This included all gene sets containing the words “differentiation” or “stem”. The resulting list of JQ1-deregulated gene transcripts was then scrutinized to identify cancer stemness-related markers.

### Quantitative RT-PCR

RNA isolation of all samples (MDA-MB-231 or BT-549 adherent, or spheroid cultures and MDA-MB-231 spheroid-derived tumours) was performed using a RNeasy Mini kit (Qiagen), as indicated above. RNA concentrations and purities were determined using a NanoDrop ND-1000 spectrophotometer (Thermo Fisher Scientific Inc.). Next, 1 μg of total RNA was reverse transcribed using a RevertAidHMinus First Strand cDNA synthesis kit (Thermo Fisher Scientific Inc.) in a thermocycler (Bio-Rad) under the following reaction conditions: 65 °C for 5 min, 42 °C for 60 min, and 70 °C for 10 min. The resulting cDNAs were subjected to quantitative real-time PCR (qRT-PCR) analysis using a Fast SYBR Green Master Mix (Thermo Fisher Scientific Inc.) in a StepOnePlus Real-Time PCR system (Applied Biosystems, Thermo Fisher Scientific Inc.). The conditions used included an initial step at 95 °C for 10 min, followed by 40 cycles at 95 °C for 15 s and a final step at 60 °C for 1 min. Each sample was analysed in triplicate, and cycle threshold (Ct) values of transcripts were determined using StepOne Software v.2.1. Ct values were calculated using *GAPDH* as reference. Untreated samples were used as controls to determine the relative fold-changes in messenger RNA (mRNA) expression. The primer sequences are listed in Supplementary Table 1.

### Immunofluorescence assays

MDA-MB-231-derived spheroids were treated with JQ1 (200 nM) and 72 h later floating spheroids were collected and gently dropped on a water repelling circle drawn on a poly-lysine-treated slide (DAKO). Within one minute, excess liquid was removed and slide-attached spheroids were fixed for 15 min with paraformaldehyde (4%). After a PBS wash, samples were blocked with Bovine Serum Albumin (BSA) (5%) for 30 min and, next, incubated with R-phycoerythrin (PE)-coupled *CD44* Antigen (CD44) (R&D Systems) for 60 min (3% BSA). Extensive washes with PBS were performed before mounting with Fluoroshield (Sigma Aldrich, Thermo Fisher Scientific Inc.). 4,6-diamidino-2-phenylindole (DAPI) (Sigma Aldrich) was used for the staining of nuclei. Fluorescence imaging of spheroids was performed using confocal microscopy (Zeiss LSM 710).

### Limiting dilution and secondary and tertiary tumour sphere formation assays

For the limiting dilation assays (LDA), MDA-MB-231 and BT-549 primary tumour spheres (TS), obtained from 21-day old spheroids, were mechanically disaggregated and counted before being seeded in non-adherent 96-well plates (Corning). Next, serial dilutions were performed to achieve 200 cells/well down to 1 cell/well. Spheroid formation was monitored until day 21 by determining the number of TS per well.

For the secondary and tertiary TS formation assays, primary TS were mechanically dissociated after which 200.000 cells were cultured in ultralow attachment 100 mm plates (Falcon) in the presence of JQ1 (200 nM) for 3 days. Next, the number of secondary TS per plate were determined. Secondary TS were again dissociated and left in the presence of the drug until day 6, at which the number of tertiary TS per plate was determined. TS pictures were taken at the two time points using an inverted microscope (Nikon).

### Matrigel invasion assays

Cells from freshly dissociated MDA-MB-231 and BT-549 spheroids (10,000 cells) were seeded on a thin layer of matrigel in 48-well plates. After overnight incubation, the cells were exposed to JQ1 (200 nM), and 3 days later 3D invading structures were visualized under an inverted Nikon Eclipse TS1000 (20x) optical microscope.

### Flow cytometry assays

For cell cycle analyses, MDA-MB-231 TS (500,000 cells) were cultured for 24 h before exposure to JQ1 (100 and 200 nM) for 1 day. Floating cells were collected and washed twice with cold PBS, fixed in ice-cold 70% ethanol for 30 min and, next, centrifuged at 5000 rpm for 5 min. The resulting cell pellets were washed in PBS containing 2% BSA to prevent cell aggregation prior to incubation with propidium iodide (PI)/RNAse staining solution (Immunostep S.L., Salamanca, Spain) in the dark for 1 h at 4 °C. Next, the samples were analysed on a FACSCanto II flow cytometer (BD Biosciences) and the percentage of cells in each cell cycle phase was determined by plotting spheroid DNA content against cell number using FACS Diva software.

For apoptosis evaluations, MDA-MB-231 TS (300,000 cells) were cultured for 24 h before exposure to JQ1 (100 and 200 nM). Three days later, floating cells were collected, centrifuged (900 rpm, 5 min), and washed twice with ice**-**cold PBS before staining in 5 ml Annexin V/DT-634 (Immunostep S.L.) in 1x binding buffer (10 mM HEPES, pH 7.4, 140 mM NaOH, 2.5 mM CaCl_2_) for 1 h in the dark at room temperature. Viable cells, as indicated by negative Annexin V staining, were determined using a FACSCanto II flow cytometer (BD Biosciences).

### In vivo studies

Balb/c nude mice (female, 6 weeks, *n* = 6/group) were orthotopically injected with freshly dissociated MDA-MB-231-derived spheroids (2 × 10^6^ cells). 14 days later, when the tumours reached ~250 mm^3^ (Tumour volume = (length x width^2^)/2), the mice were treated daily for three days with JQ1 (50 mg/kg, intravenously). Next, the tumours were harvested and cryogenically stored until qRT-PCR analyses were performed (see above). All animal studies were carried out according to protocols approved by the ethics committee on animal experimentation of Castilla-La Mancha University (Procedure PR-2017-03-07).

### Clinical outcome analyses

The Kaplan-Meier (KM) Plotter Online Tool was used to analyse relationships between the expression of JQ1-deregulated stemness genes and TNBC patient clinical outcome (http://kmplot.com/analysis/, Relapse Free Survival (RFS), basal-like breast cancer; data accessed: 11/04/18). By using this tool, the effect of 54,675 genes on survival was assessed using 5143 breast cancer samples. For the analyses, patients were separated according to best cut-off values (expression range of the signature = 170–11,816), and the following probes were used: Epithelial Adhesion Molecule (*EPCAM)* (201839_s_at), SRY-related HMG-box gene 9 (*SOX9)* (202936_s_at), Integrin α6 (*ITGA6)* (201656_at), Gap Junction Protein Alpha 1 (*GJA1)* (201667_at), V-Myc avian myelocytomatosis viral oncogene homolog (*MYC)* (202431_s_at), *ABCG2* (209735_at), *EZH2* (203358_s_at), Runt-related transcription factor 2 (*RUNX2)* (232231_at), Follistatin Like 1 (*FSTL1)* (208782_at), Protein Inhibitor of Activated STAT 3 *(PIAS3)* (203035_s_at), Aldehyde Dehydrogenase 1 Family Member A1 (*ALDH1A1)* (212224_at), *CD24* Antigen (*CD24)* (266_s_at), and *CD44* (212063_at). Only deregulated genes significantly associated with detrimental outcome (Hazard Ratio (HR) > 1 and *p* value ≤ 0.05) were used for subsequent analyses (*n* = 4). This tool was also used to determine RFS in combined analyses of the four poor prognosis-linked genes. All the analyses were performed independently by two authors (SMG and MNC) and reviewed by a third author (EMGM). No discrepancies were noted.

We established a database of transcriptomic datasets with available response and treatment data to study the relationship between JQ1-deregulated stemness gene expression and clinical outcome (RFS) regarding the chemotherapy regimen followed (5 years post-treatment). Through a Pubmed search using the keywords “breast cancer”, “survival”, “treatment”, and “response”, we identified 2108 breast cancer cases that received chemotherapy and for whom the gene expression levels were measured using Affymetrix HGU133A and HGU133A plus 2.0 microarrays. Gene expression levels were studied for patients who received chemotherapy with Taxane, Anthracycline, Ixabepilone, CMF (Cyclophosphamide / Methotrexate / Fluorouracil), FAC (Fluorouracil / Adriamycin / Cytoxan), and FEC (Fluorouracil / Epidoxorubicin / Cyclophosphamide).

### BETi-associated stemness interactome analysis

The online tool STRING (http://www.string-db.org) was used to construct an interactome map of JQ1 stemness deregulated genes (STRING v10, data accessed: 14/03/18). *Node degree:* average number of interactions; *Clustering coefficient:* indicates the tendency of the network to form clusters. The closer the local clustering coefficient is to 1, the more likely it is for the network to form clusters; *PPI enrichment p value:* indicates the statistical significance.

### Statistical analysis

All in vitro experiments were performed at least three times and, except for the flow cytometry studies, each condition was prepared in triplicate. Two-way ANOVA and Student’s test were used for the statistical analyses (*p* < 0.05*, *p* < 0.01**, *p* < 0.001***).

## Results

### JQ1 modifies the expression of stemness-related genes

**Fig. 1:**
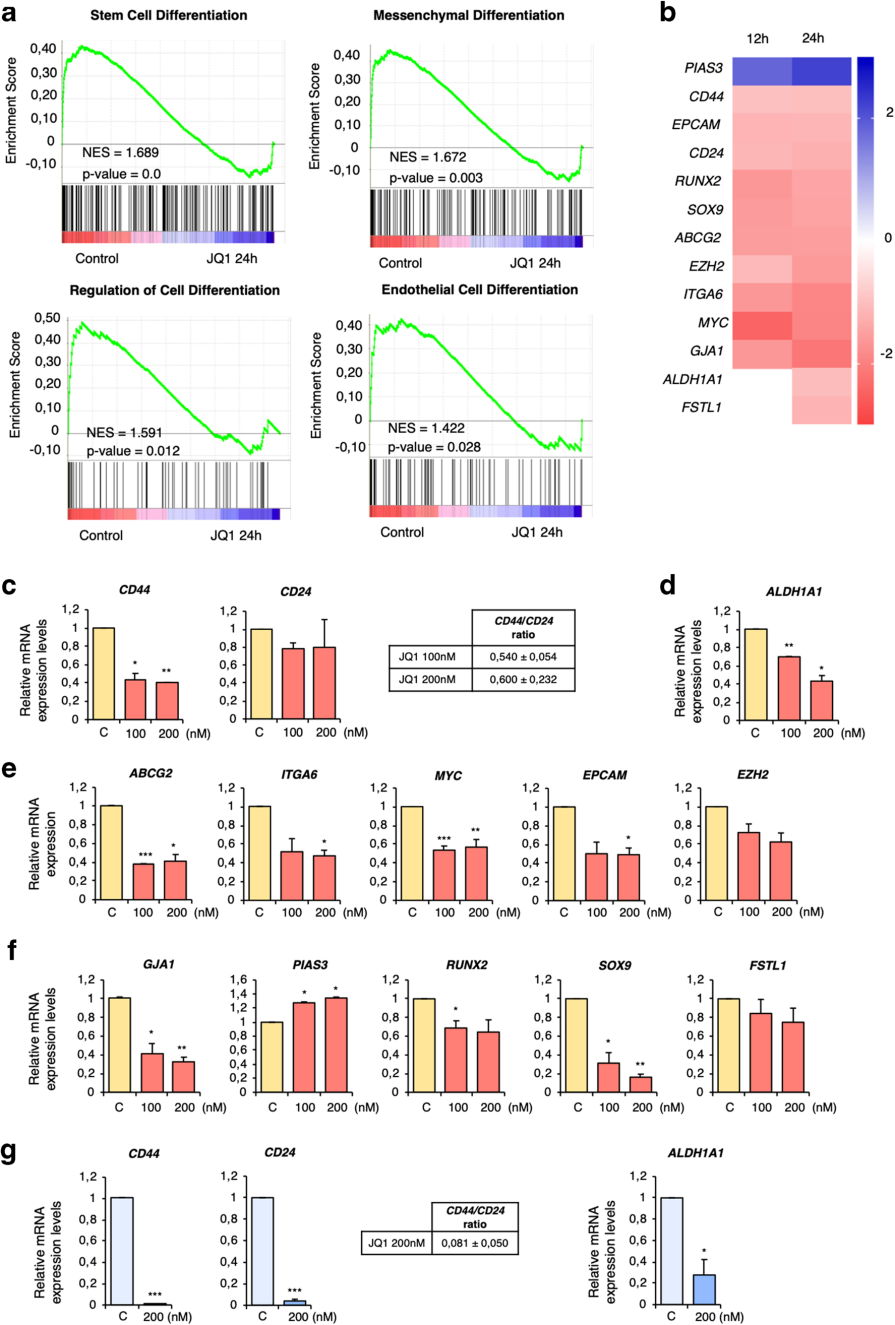
JQ1 affects stemness-related functions and downregulates the expression of stemness-related genes. **a**. Gene Set Enrichment Analysis (GSEA) identifying stemness-related biological functions. **b**. Manual screening uncovering 13 stemness-related genes. Heat map indicating fold changes of JQ1-deregulated genes compared to those in non-treated cells (incubated with vehicle). **c-g.** JQ1 effect on mRNA levels of the stemness-related genes identified in **b** corroborated by qRT-PCR. MDA-MB-231 (**c-f**) or BT549 (**g**) cells were treated with the indicated doses of JQ1 (24 h) after which qRT-PCR was performed for the indicated genes. **c** and **g**. *CD44* and *CD24* mRNA levels were used to calculate the *CD44/CD24* ratios in JQ1 exposed cells compared to control cells**. d** and **g***ALDH1A1* mRNA levels were significantly downregulated in cells treated with JQ1 at both doses. **e.** JQ1 significantly decreased *ABCG2, ITGA6, MYC* and *EPCAM* levels in MDA-MB-231 cells, and modestly affected the *EZH2* level. **f.***GJA1, PIAS3, RUNX2* and *SOX9* mRNA levels were significantly altered by JQ1, while just a slight decrease was found for *FSTL1*. The qRT-PCR conditions are described in material and methods. The results shown are averages of 3 independent experiments performed in triplicate. Student t-test: * *p* < 0,05, ** *p* < 0,01 and *** *p* < 0,001

First, molecular functions modified by BET inhibition were explored at the transcriptomic level, with a special focus on those related to stemness. To this end, MDA-MB-231 cells were treated with JQ1 for 12 or 24 h. Next, mRNA profiling analysis was performed using an Affymetrix Transcriptome Array 2.0. In total 4652 and 2530 deregulated transcripts were found at 12 and 24 h, respectively. A preliminary analysis performed with the functional annotation tool DAVID revealed “Regulation of cell differentiation” as one of most ranked deregulated functions upon JQ1 treatment (Supplementary Fig. 1). Further functional enrichment analyses of the latter time point using GSEA software confirmed a decrease in several stem-related functions, including cell differentiation, stem cell differentiation, mesenchymal differentiation, regulation of cell differentiation, and endothelial cell differentiation (Fig. 1a and Supplementary Table 2).

Next, the list of deregulated transcripts was screened for genes known to be directly involved in stemness. This search uncovered a panel of 13 stem-related deregulated genes at 12 h and 13 genes at 24 h (Fig. 1b). The microarray-based results were corroborated by qRT-PCR, and we found that even low doses of JQ1 had an impact on the mRNA expression levels of the identified genes (Fig. 1c-g). To confirm its effect on the CSC population, the *CD44/CD24* ratio and the expression of *ALDH1A1*, both well-known CSC markers [21], were examined. We found that treatment with JQ1 induced decreases in the *CD44/CD24* ratio (Fig. 1c and g) together with a significant reduction in *ALDH1A1* expression levels (Fig. 1d and g). Additionally, downregulation of the stemness-related genes *ABCG2, ITGA6, MYC, EPCAM* and *EZH2* (Fig. 1e) and other well-known stemness-associated genes, such as *GJA1, RUNX2, SOX9* and *FSTL1,* identified in the microarray screen, were confirmed to be downregulated upon JQ1 exposure by qRT-PCR, together with an increase in expression of the stemness repressor *PIAS3* (Fig. 1f). These data confirm the impact of JQ1 on cancer stemness and identify a panel of stemness genes associated with its application.

### BET inhibition impairs stem cell-like features in TNBC spheroids

**Fig. 2:**
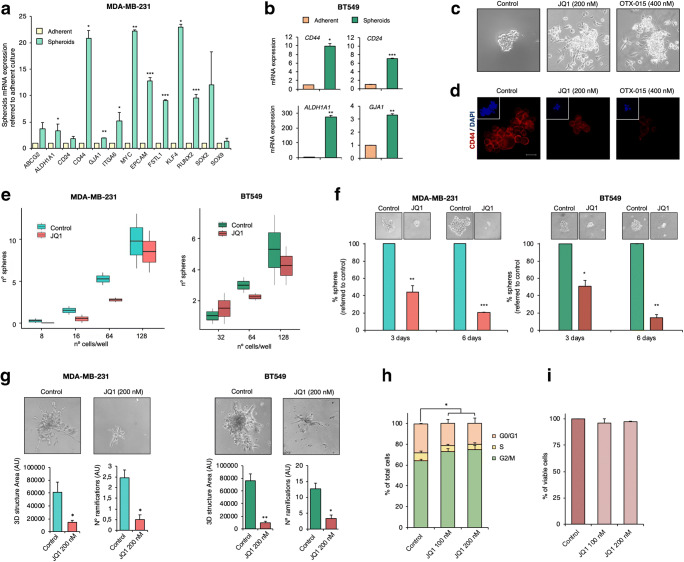
BET inhibitor JQ1 impairs crucial stemness-related functions in CSC-mimicking spheroid models. **a**. Relative mRNA expression levels of several CSC markers in the MDA-MB-231 spheroid model (green) compared to those in adherent cultures (light yellow). **b.** Relative mRNA expression levels of *CD44, CD24, ALDH1A1* and *GJA1* in the BT-549 spheroid model (dark green) compared to those in adherent cultures (orange). **c.** MDA-MB-231 cells were treated with the JQ1 or OTX-015 at the indicated doses for 72 h after which images were taken using an inverted microscope. **d.** Remaining floating spheres from **c** were tested for CD44 surface expression (Red). Stained spheroids were observed by confocal microscopy. Squares display TS nuclear counterstaining with DAPI (blue). **e.** MDA-MB-231 and BT549 spheroids were mechanically dissociated and next exposed to JQ1 (200 nM). Secondary formed spheroids were counted at day 3 and, subsequently, dissociated again to produced tertiary spheroids (day 6). Pictures were taken at both time points using and inverted microscope. **f.** MDA-MB-231 and BT549 spheroids were mechanically dissociated after which serial dilutions were performed to cover a range from 1 to 200 cells per well (in triplicates). LDA in the absence and presence of JQ1 (200 nM) was performed to evaluate the self-renewal capacity of the spheroid models. The ability to form tumour spheres in both conditions was evaluated at day 21 and formed spheroids were counted. **g.** Freshly dissociated MDA-MB-231 or BT549 spheroids (10.000 cells/well in 48-well plates) were gently layered on a thin matrigel matrix. 24 h later, matrigel-embedded cells were left untreated or exposed to JQ1 (200 nM). 48 h later, pictures of the invasion structures were taken, and 3D structure areas and the number of ramifications of each condition were evaluated. **h.** MDA-MB-231 spheroids were incubated with JQ1 at the indicated doses. After 24 h, cell cycle progression was examined by flow cytometry. DNA staining was performed using propidium iodide. The histogram shows the percentage of cells in the different phases of the cell cycle**. i.** MDA-MB-231 spheroids were exposed to the indicated doses of JQ1. After 72 h, apoptotic activity was assessed by flow cytometry by Annexin V binding evaluation. The histogram shows the percentage of viable cells (Annexin V-). All results shown represent the average of 3 independent experiments. Student t-test: * *p* < 0,05, ** *p* < 0,01 and *** *p* < 0,001

Previously, three-dimensional cell culture models have been shown to be of use for cancer research [22]. As a general rule these models, grown under particular conditions, are enriched in stem cell markers, turning them into good models to explore the effects of BETi on stemness. Thus, to further confirm that MDA-MB-231 and BT-549 spheroids can be employed as in vitro models for CSCs, the basal expression of the genes identified in the transcriptomic analyses were compared in the spheroids and the parental adherent cells. Marked increases in the mRNA levels of these stemness markers were observed in the spheroid cultures (Fig. 2a-b). Of note, in comparison to the adherent cells, the *CD44/CD24* ratio was increased in the two spheroid models, being more than ten-fold in the MDA-MB-231 spheroid model (*CD44/CD24* ratio = 11.48). Notably, *ALDH1A* expression was also augmented in both 3D models, especially in the BT549 spheroid model. The expression levels of other stem cell markers, such as Kuppel Like Factor 4 (*KLF4*) and SRY-Box2 (*SOX2*)*,* were also found to be enhanced in the spheroid models (Fig. 2a).

Next, the effect of JQ1 on spheroid growth was explored. We found that exposure to JQ1 provoked a loss of the ability to grow in suspension, leading to the attachment of the spheroids to the culture plate surface, an indication of loss of stemness (Fig. 2c). Similar results were obtained with the BETi OTX-015 (Fig. 2c). The remaining spheroids were assessed for CD44 expression by immunofluorescence. We found that JQ1 induced a marked decrease in CD44 expression (Fig. 2d). To next evaluate the impact of JQ1 on MDA-MB-231 and BT-549 spheroid self-renewal capabilities, a limiting dilution assay (LDA) in the presence of the drug was carried out. In support of a negative effect of JQ1 on stemness, cells exposed to JQ1 exhibited a reduced capacity to form primary spheres (Fig. 2e). To further investigate its influence on this stemness hallmark, secondary and tertiary TS formation assays were performed. We found that treatment with JQ1 drastically reduced the number and size of the spheres in both models (Fig. 2f). Another stem cell-like feature, i.e., invasion potential, was investigated via 3D matrigel assays using MDA-MB-231 and BT-549 spheroids. In agreement with the previous results, we found that JQ1 impaired the invasive ability of spheroids, resulting in a decrease of the area of the 3D structures formed and a reduction of the number of ramifications (Fig. 2g). Finally, in line with previous results in MDA-MB-231 adherent cells [12], cell cycle analyses by flow cytometry revealed that JQ1 also induced G0/G1 arrest in spheroids (Fig. 2h), without any impact on cell death (Fig. 2i).

### Expression of stemness-related genes in JQ1-treated spheroids and xenograft tumours

**Fig. 3:**
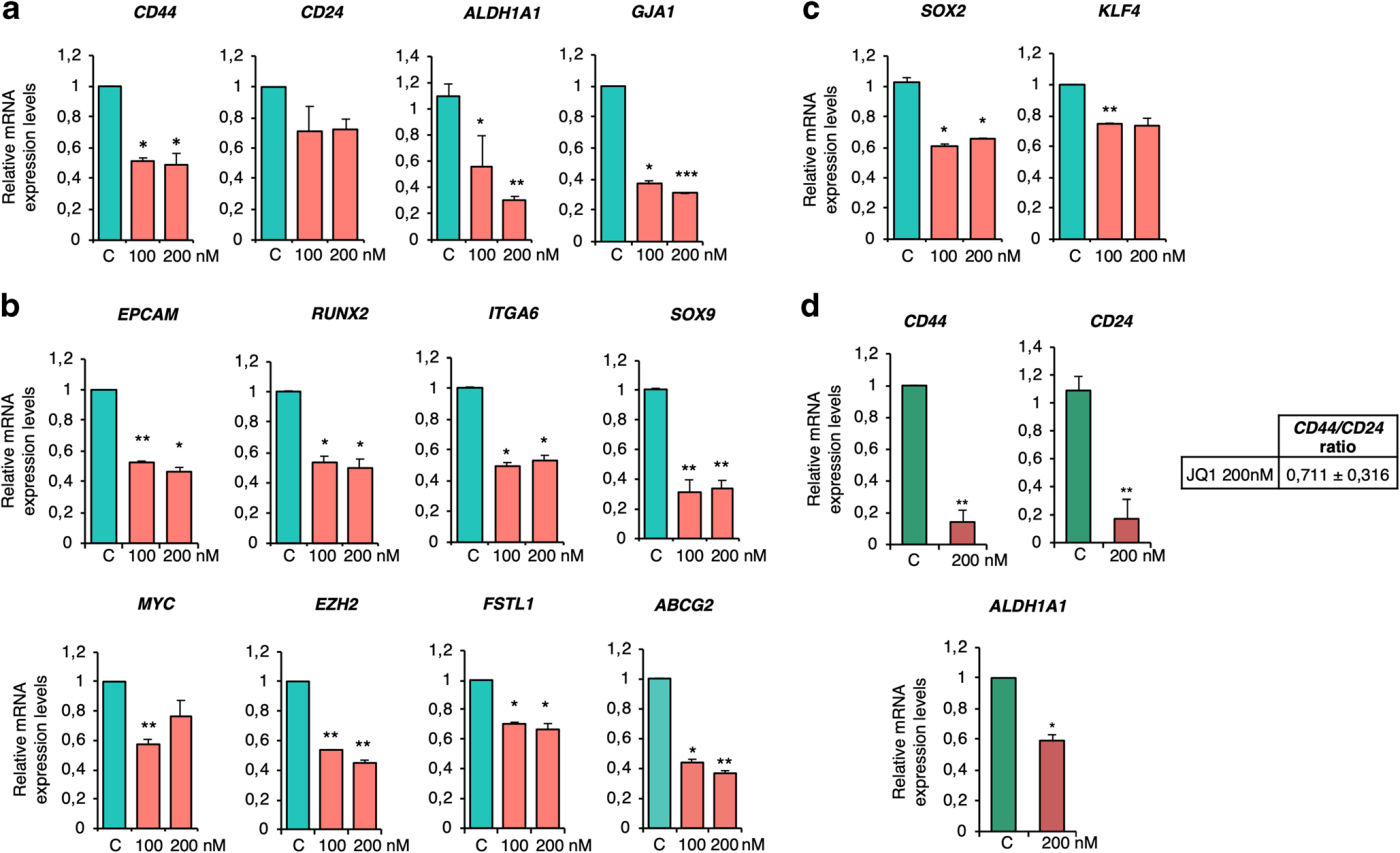
JQ1 efficiently decreases the expression of CSC markers in MDA-MB-231 (**a**) and BT549 (**b**) spheroids. **c**. Effect of JQ1 on the classical stemness markers *SOX2* and *KLF4*. **d**. Relative mRNA levels of *CD44, CD24 and ALDH1A* in spheroids exposed to JQ1 in MDA-MB-231 and BT549 spheroids, respectively. The results shown represent the average of 3 independent experiments performed in triplicate. Student t-test: * *p* < 0,05, ** *p* < 0,01 and *** *p* < 0,001

Next, we aimed to confirm the microarray-based findings using validated CSC-mimicking models. To this end, the effect of JQ1 on the expression of the identified stem cell-like and CSC-like genes was investigated in MDA-MB-231 and BT-549 spheroids. First, the impact of JQ1 on *CD44, CD24* and *ALDH1A1* expression was evaluated. JQ1 treatment led to a marked decrease of the *CD44/CD24* ratio (0.74 ± 0.13 and 0.68 ± 0.03 for 100 and 200 nM, respectively, in the MDA-MB-231 model, and 0.71 ± 0.09 for 200 nM in the BT-549 model), together with a dramatic decrease in *ALDH1A1* expression (Fig. 3d). The level of the stem cell marker *GJA1*, which is known to be enriched in CD44+ populations, was also reduced upon JQ1 exposure in the MDA-MB-231 model (Fig. 3a). Treatment with JQ1 efficiently downregulated the expression of the established CSC markers *EPCAM, RUNX 2 and ITGA6,* as well as the levels of other CSC-related genes, such as *MYC, EZH2, FSTL1* and *ABCG2* (Fig. 3b). The BETi also decreased the mRNA expression levels of the identified TS-enriched classical stemness markers *SOX2* and *KLF4* (Fig. 3c).

**Fig. 4:**
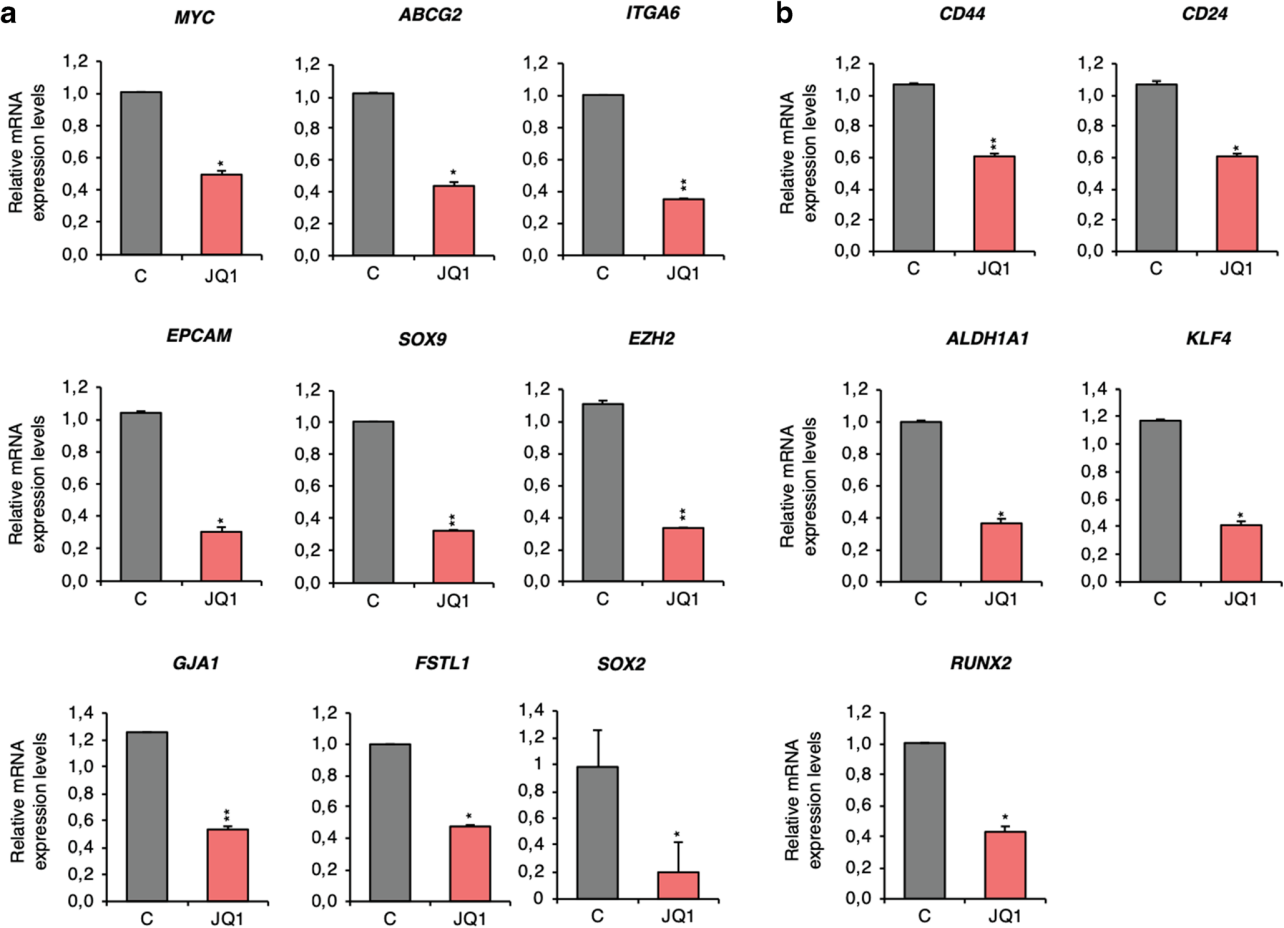
JQ1 effects the expression of CSC markers in an in vivo pre-clinical model. **a**. *MYC, ABCG2, ITGA6*, *EPCAM, SOX9, EZH2, GJA1, FSTL1* and *SOX2* mRNA levels in JQ1-treated tumours compared to vehicle-treated tumours. **b**. *CD44*, *CD24, ALDH1A1, KLF4 and RUNX2* mRNA level changes observed upon JQ1 exposure. The qRT-PCR conditions are described in material and methods. Student t-test: * *p* < 0,05, ** *p* < 0,01 and *** *p* < 0,001

To test the potential of JQ1 to control the identified stemness-related gene panel in a more physiological context, its effect on MDA-MB-231 spheroid-derived xenografts was evaluated. First, Balb/c nude mice (female, 6 weeks) were injected with freshly dissociated MDA-MB-231 spheroids. Two weeks later, tumour-bearing mice were treated with JQ1 during three consecutive days. Having completed the treatment regimen, the mRNA levels of the identified panel of genes were analysed in the collected tumours. Our results revealed that JQ1 also affects the expression of *MYC, ABCG2, ITGA6, EPCAM, SOX9, EZH2,* GJA1, *FSTL1* and *SOX2* in this in vivo model (Fig. 4a). It had also a negative effect on the expression of *CD24, CD44, ALDH1A, KLF4 and RUNX2* (Fig. 4b), further supporting a potential role for this BETi to alter stemness.

### JQ1-associated stemness signature predicts a worse patient outcome

**Fig. 5:**
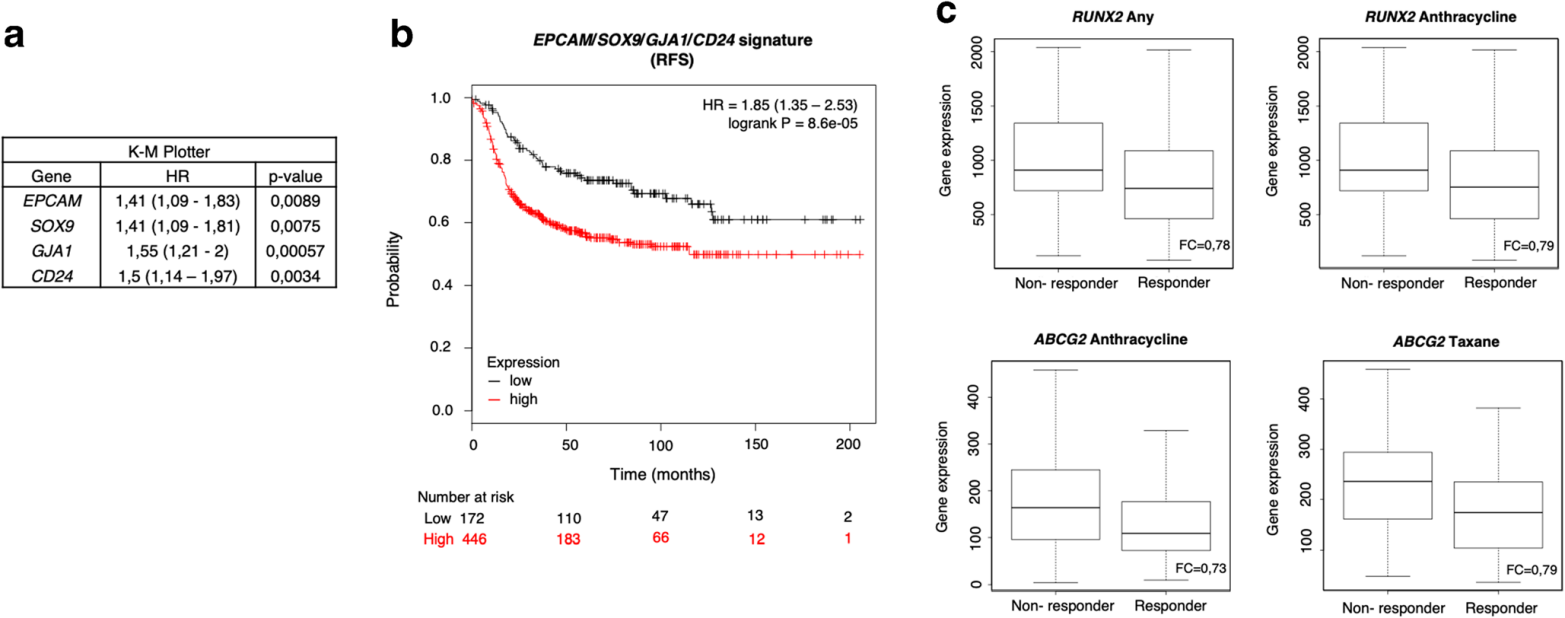
JQ1-controlled genes *EPCAM*, *SOX9*, *GJA1* and *CD24* correlate with a poor prognosis, while *RUNX2* and *ABCG2* correlate with worse response to chemotherapy. **a**. The Kaplan-Meier plotter online tool was used to assess potential relations of JQ1 downregulated stemness-related genes and disease progression in basal-like breast cancer patients. Hazard Ratio (HR) and *p* values of the *EPCAM, SOX9, GJA1* and *CD24* genes with a significant association a with worse relapse-free survival (RFS) are listed in the table. The cut-off values used to separate patients, referred to as best cut-off, were 8464, 2557, 3196 and 4844, respectively. **b**. Using the same tool, a combined analysis with the four genes to assess their link to patient prognosis was performed. Kaplan-Meier plots compare tumours with a low combined expression of these four genes with those exhibiting a high combined expression. HR and logrank *p* values are indicated. The selected cut-off value = 3733.5. **c**. Using transcriptomic data of 2108 patients with available treatment and response data, potential associations between 13 JQ1-controlled stemness-related genes and patient responses to chemotherapy were evaluated. The *ABCG2* and *RUNX2* mRNA expression levels in responder and non-responder patients, measured as RFS five years after chemotherapy completion, are shown for several chemotherapy regimens

To evaluate the role of the identified genes in tumour relapse, we decided to study their association with clinical outcome in TNBC. Using data contained in the Kaplan Meier (KM) plotter online tool [23], we found that high expression levels of *GJA1, CD24, EPCAM* and *SOX9* correlated with a poor recurrence free survival (RSF) (HR = 1.55 (1.21–2), *p* value = 0.00057; HR = 1.5 (1.14–1.97), *p* value = 0.0034; HR = 1.41 (1.09–1.83), *p* value = 0.0089; HR = 1.41 (1.09–1.81), *p* value = 0.0075, respectively) (Fig. 5a). Combined analysis of these four poor prognosis-associated genes revealed a higher potential to predict patient outcome than each gene individually (HR = 1.85 (1.35–2.53), log-rank *p* = 8.6e-05) (Fig. 5b). No association with clinical outcome was observed for the remaining genes.

Next, potential correlations between the identified genes and patient responses to antitumor therapy were assessed. To do so, correlations between responses to therapy and gene expression in over 2108 patient samples were determined. High mRNA levels of the JQ1-altered stemness-related genes *ABCG2* and *RUNX2* were found to correlate with poorer responses to chemotherapy (Fig. 5c, Supplementary Fig. 2). Specifically, high levels of *RUNX2* expression were associated with low responses to any chemotherapy (Taxane, Anthracycline, Ixabepilone, CMF (Cyclophosphamide / Methotrexate / Fluorouracil), FAC (Fluorouracil / Adriamycin / Cytoxan), and FEC (Fluorouracil / Epidoxorubicin / Cyclophosphamide). On the other hand *ABCG2*, a well-studied multi-drug resistance-associated gene [24], showed correlations with only two chemotherapies (Taxane and Anthracycline). Therefore, *RUNX2* may serve as a universal biomarker to predict chemotherapy response.

## Discussion

The stemness landscape may define tumour aggressiveness through its effect on tumour progression and response to treatment. Accordingly, major interest in the study and measurement of stemness characteristics in cancer has recently evolved [8, 25] aimed at the development of novel strategies to target this feature. In breast cancer, stemness is a common characteristic that has been strongly associated with molecular and clinical features, especially in the TNBC subtype [9]. Indeed, TNBC is characterized by an enriched population of CSCs that confer intrinsic therapy resistance and aggressiveness [4, 20]. Therefore, the identification of drugs targeting CSCs may be a promising approach to treat TNBC.

BETi have recently emerged as novel epigenetic cancer treatment options, exhibiting pre-clinical efficacy in several malignancies, including acute myeloid leukaemia and breast cancer [15, 26]. In early stage clinical studies, good tolerability and signs of clinical activity for some of these compounds have been shown [15]. Among the different mechanisms of action, BETi have been found to play a role as regulators of self-renewal and stem cell signalling in some cancers, including medulloblastoma and head and neck squamous cell carcinoma [27, 28]. Although several studies have investigated the impact of BET protein inhibition in breast cancer [29–33], some of them evaluating its impact on epithelial-to-mesenchymal transition [34], little is known about the impact of their associated epigenetic drugs on cancer stemness in this cancer type. Here, we show how the BETi JQ1 alters the genetic stemness landscape, together with its associated features, and identify a stemness-related gene panel associated with BET inhibition in TNBC. By using different parameters to evaluate biomarkers of cancer stemness, such as overexpression of CSC markers, elevated *CD44/CD24* ratios, self-renewal capabilities and high invasion potentials, we found that JQ1 can jeopardize stemness in TNBC.

First, our transcriptomic analysis and qRT-PCR results in 2D, 3D and in vivo models, revealed several JQ1-associated stemness-related genes in TNBC, including *CD44, CD24* and *ALDH1A1*. The *CD44/CD24* ratio, as well as *ALDH1A1* over-expression, are known indicators of tumour aggressiveness, correlating with high proliferation indexes, increased tumour initiation capabilities and augmented invasion and metastasis potentials [21]. We found that JQ1 led to an almost 50% decrease in the *CD44/CD24* ratio and an up to 70% decrease in *ALDH1A1* expression in TNBC cells. We also found that JQ1 negatively affected several transcription factors known to be involved in the initiation and maintenance of the stemness phenotype in TNBC, such as *MYC, EZH2, SOX9* and *RUNX2* [35–37]. *MYC, EZH2* and *SOX9* are known to be repressed in response to BET inhibition in several malignancies, such as medulloblastoma, bladder cancer and myelofibrosis [14, 38–40], but as yet the effect of JQ1 on these genes in TNBC has not been explored. Moreover, we found that *RUNX2,* a stemness marker highly expressed in TNBC [41] and known to play a crucial role in breast cancer metastasis [42], can be negatively targeted by BETi in TNBC. This result, which is supported by previous data [43], could open the way to the development of RUNX2-targeting therapies. On the other hand, JQ1 may deregulate stemness genes that code for proteins involved in several other functions, such as EPCAM, ABCG2, ITGA6, GJA1, FSTL1 and PIAS3. These proteins have been linked to cancer progression, resistance to chemo- and radiotherapy, poor prognosis and/or metastasis [4, 44–51]. Therefore, targeting these proteins with JQ1 could be of clinical interest in TNBC.

Two-dimensional cell culture models of cancer have progressively lost support over the last few years in favour of 3D models, which better reflect the in situ behaviour of cancer cells [22]. Several tools have been used to develop in vitro 3D cultures that mimic stemness features and are capable of predicting toxicity and resistance to anti-tumour compounds. Here, we used a MDA-MB-231-derived spheroid model to recapitulate TNBC and demonstrate that JQ1 not only modulates stemness-related genes in this 3D model, but also represses tumour self-renewal and invasion potentials, which are crucial stemness characteristics. JQ1 treatment did not lead to apoptosis in this population, but instead induced an increase in the G0/G1 population, indicative of cell cycle arrest in this phase. More importantly, we found that his BETi directly impacted tumour sphere formation, a key stemness feature, as indicated by secondary and tertiary sphere formation and limiting dilution assays. This result points to a direct role of JQ1 as a tumour stemness regulator.

As mentioned above, targeting tumour stemness may be a good strategy to revert chemotherapy resistance and cancer recurrence. In this sense, some of the stemness-related genes identified in this study were able to predict relapse and a lack of response to chemotherapy in breast cancer patients. Among them, *GJA1*, *CD24*, *EPCAM* and *SOX9* were found to be associated with a worse patient prognosis. Interestingly, we also found that combined expression of these four genes had a higher predictive potential than each gene individually. This may have clinical implications, as this 4-genes subpanel may potentially serve as a TNBC prognostic biomarker set.

On the other hand, using an online transcriptome-level validation tool for predictive biomarkers [52], we found that *ABCG2,* associated with resistance to chemo- and radiotherapy [53] and *RUNX2*, which has been shown to chemo-sensitize breast cancer cells to neoadjuvant therapy [43], are more highly expressed in non-responder patients. Although not all the parameters analysed reached statistical significance, the observed trends could be corroborated using other probes available in the tool (data not shown). The association of *ABCG2* with resistance to therapy has been extensively confirmed [54]. Our results also confirm a role for *RUNX2* as a potential predictive marker for chemotherapy response in TNBC. Although no significant association with either patient outcome or response to treatment was found for the rest of the genes contained in the stemness-related panel, evaluation of the expression levels of the whole stemness-panel, including all the 13 identified genes, might be of interest for the choice of treatment in patients with aggressive tumours.

Although BETi exhibit marked antitumor activities in TNBC, single treatment seems to have limited value for clinical cancer management. Conversely, combination therapy is gaining ground [55]. BETi have, for example, successfully been combined with kinase inhibitors [13, 56] and histone deacetylase inhibitors [57] in breast cancer, but its combination with chemotherapeutic agents has not been explored yet. Our data support the use of JQ1 in combination with other anticancer drugs to overcome therapy resistance.

## Conclusions

**Fig. 6:**
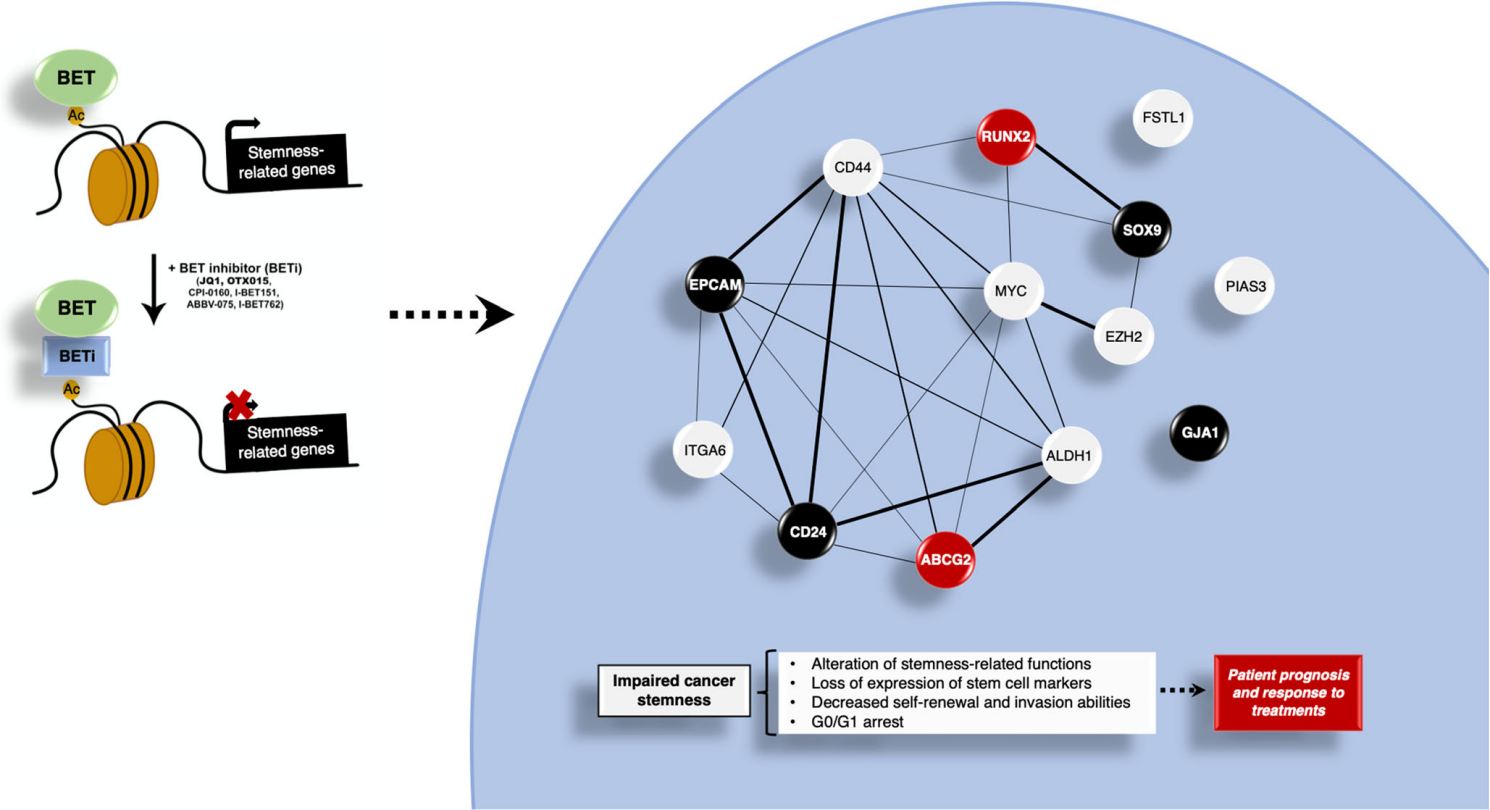
JQ1 modifies stemness landscape in TNBC. JQ1 controls stemness-related genes in TNBC, which leads to impairment of cancer stemness. Thus, this BETi alters stemness-related functions, decreases self-renewal and invasion capabilities and provokes cell cycle arrest in G0/G1. The protein-protein interaction (PPI) map shows a high grade of interaction of the corresponding proteins (Average node degree: 3.69; Avg. local clustering coefficient: 0.51; PPI enrichment *p*-value:7.28e-14). Black nodes indicate proteins encoded by genes associated with a poor RFS, and red nodes indicate proteins encoded by genes linked to poor response to conventional chemotherapy

In summary, in this work we describe a novel function for the BETi JQ1 in TNBC as a stemness-targeting drug. We identified a 13-genes stemness signature which can be deregulated by JQ1 and may have potential as a biomarker for chemotherapy treatment response and patient prognosis in TNBC. Given the high level of interaction of the predicted gene signature network, and given the fact that the identification of biomarker-interacting maps and the discovery of drug targets are main goals of future medicine [58], JQ1 may be considered as a stemness interactome-based drug (Fig. 6). Altogether, our results support the idea that targeting the epigenome with JQ1, through its impact on stemness-related genes and features, may help to overcome TNBC resistance to standard therapy. This may have potential therapeutic implications with respect to the choice of anti-tumour agents for each patient, allowing more individualized TNBC treatment.

## Electronic supplementary material

Supplementary Fig. 1 Functional enrichment analysis in MDA-MB-231 cells after incubation with JQ1 (500 nM) for 12 and 24 h using Gene-set enrichment analysis and DAVID Bioinformatics Resources 6.7. (PNG 196 kb)

High Resolution Image (TIFF 8790 kb)

Supplementary Fig. 2 ABCG2 and RUNX2 ROC plots for the indicated chemotherapy regime. (PNG 548 kb)

High Resolution Image (TIFF 5173 kb)

Supplementary Table 1 Primer sequences. (PNG 481 kb)

High Resolution (TIFF 8490 kb)

Supplementary Table 2 Summary of the GeneSets analysis reports. Size: number of genes; ES: enrichment score; NES: normalized enrichment score; NOM p-valor: nominal p value; FDR q-val: false discovery rate; FWER q-val: familywise-error rate. (PNG 739 kb)

High Resolution (TIFF 8490 kb)

Supplementary Table 3 Values of AUC, ROC p value, Mann-Whitney test, Mann- Whitney test p value and fold change indicating differences between responder (better RFS) and non-responder (worse RFS) patients based on RUNX2 and ABCG2 gene expression in basal-like breast cancer patients. (PNG 231 kb)

High Resolution (TIFF 8490 kb)

## Data Availability

All data generated and/or analysed during the current study are available from the corresponding author upon reasonable request.
