# Supplementary figures and images for: Identification of a stemness-related gene panel associated with BET inhibition in triple negative breast cancer
Source: Cell Oncol (Dordr). 2020 Mar 12;43(3):431–44. doi: 10.1007/s13402-020-00497-6 (PMC7214516; doi:10.1007/s13402-020-00497-6)

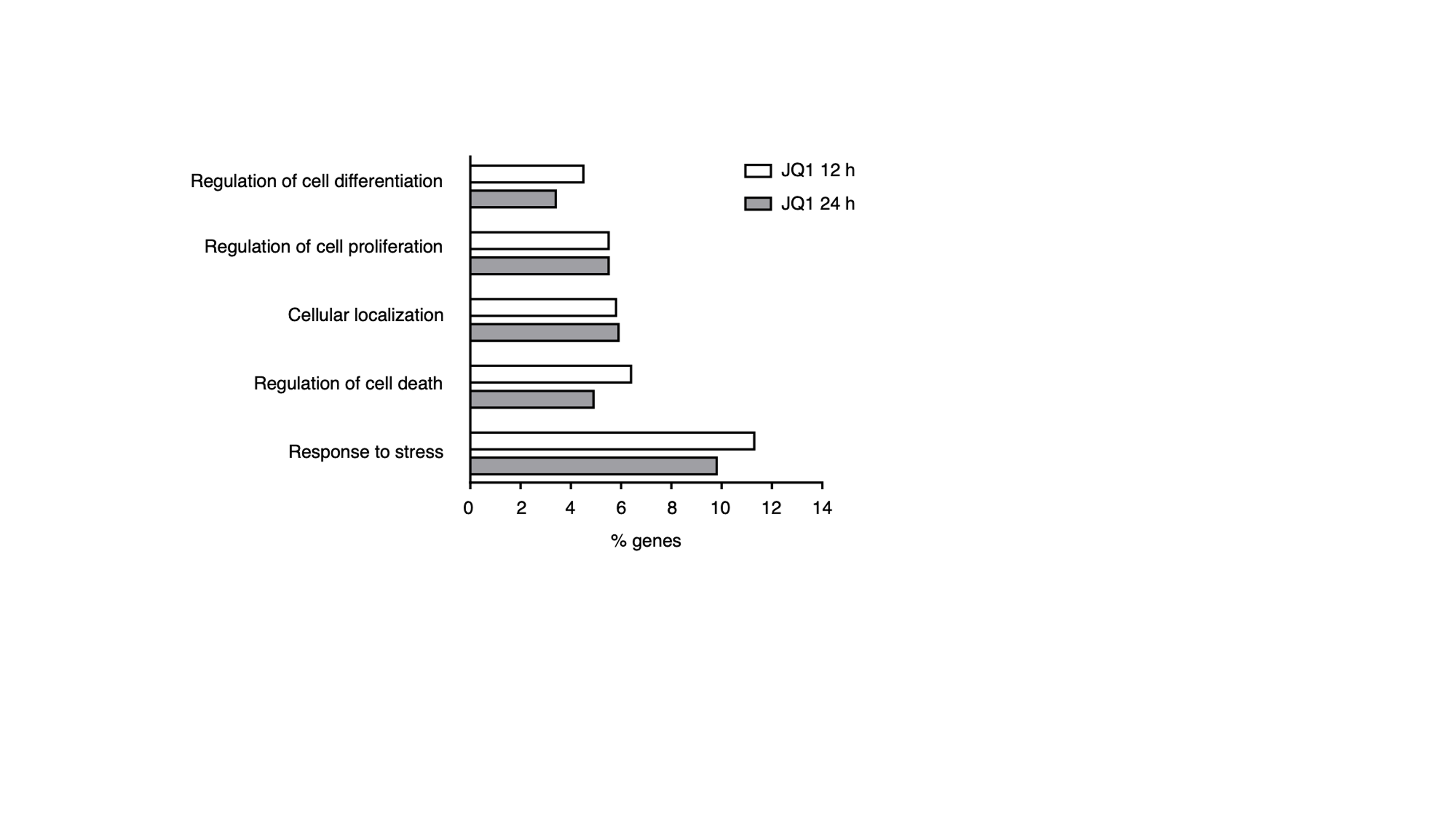

Supplement: Supplementary file 1 — Functional enrichment analysis in MDA-MB-231 cells after incubation with JQ1 (500 nM) for 12 and 24 h using Gene-set enrichment analysis and DAVID Bioinformatics Resources 6.7. (PNG 196 kb) [file 13402_2020_497_Fig7_ESM.png]

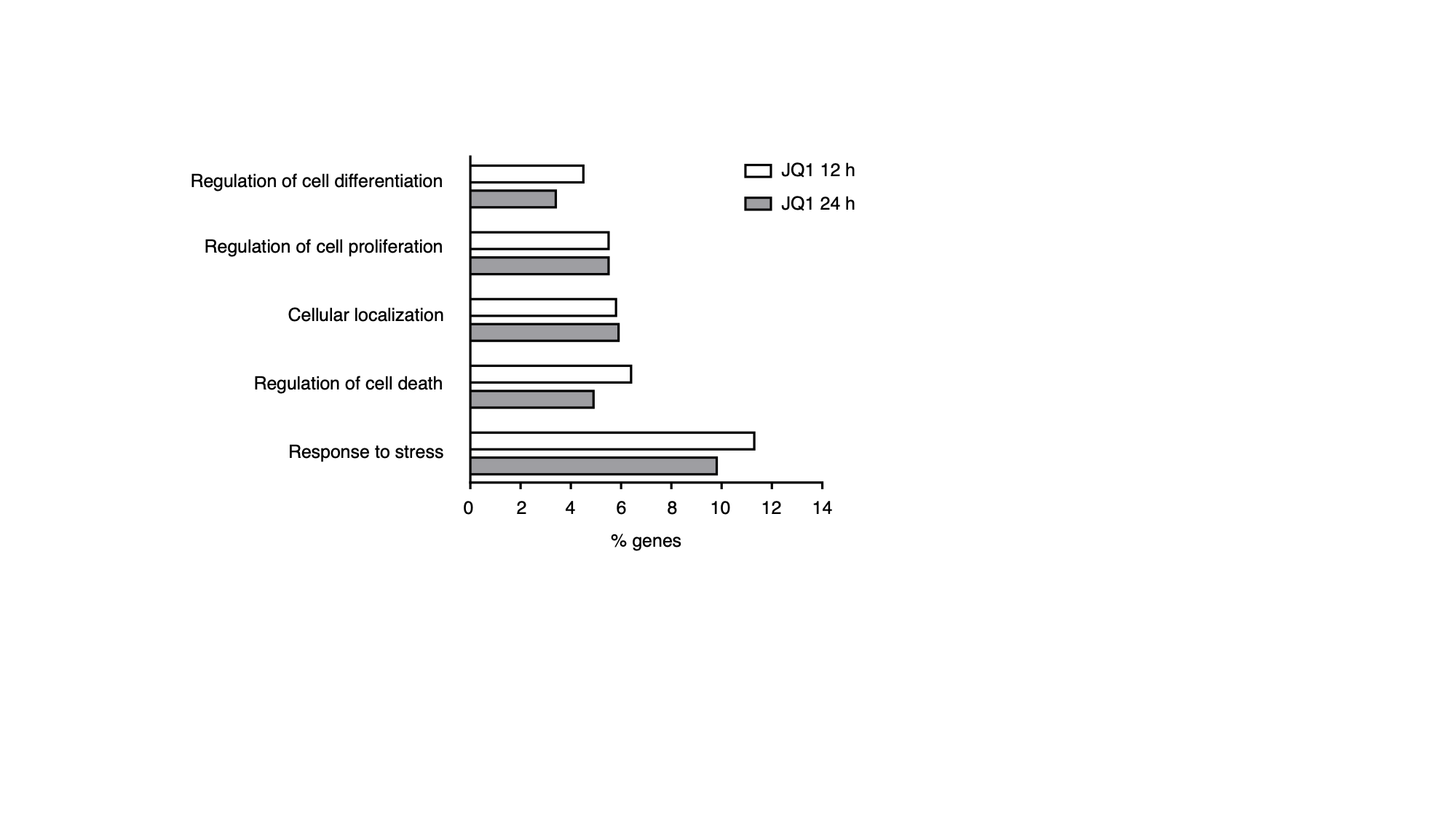

Supplement: Supplementary file 2 — High Resolution Image (TIFF 8790 kb) [file 13402_2020_497_MOESM1_ESM.tiff]

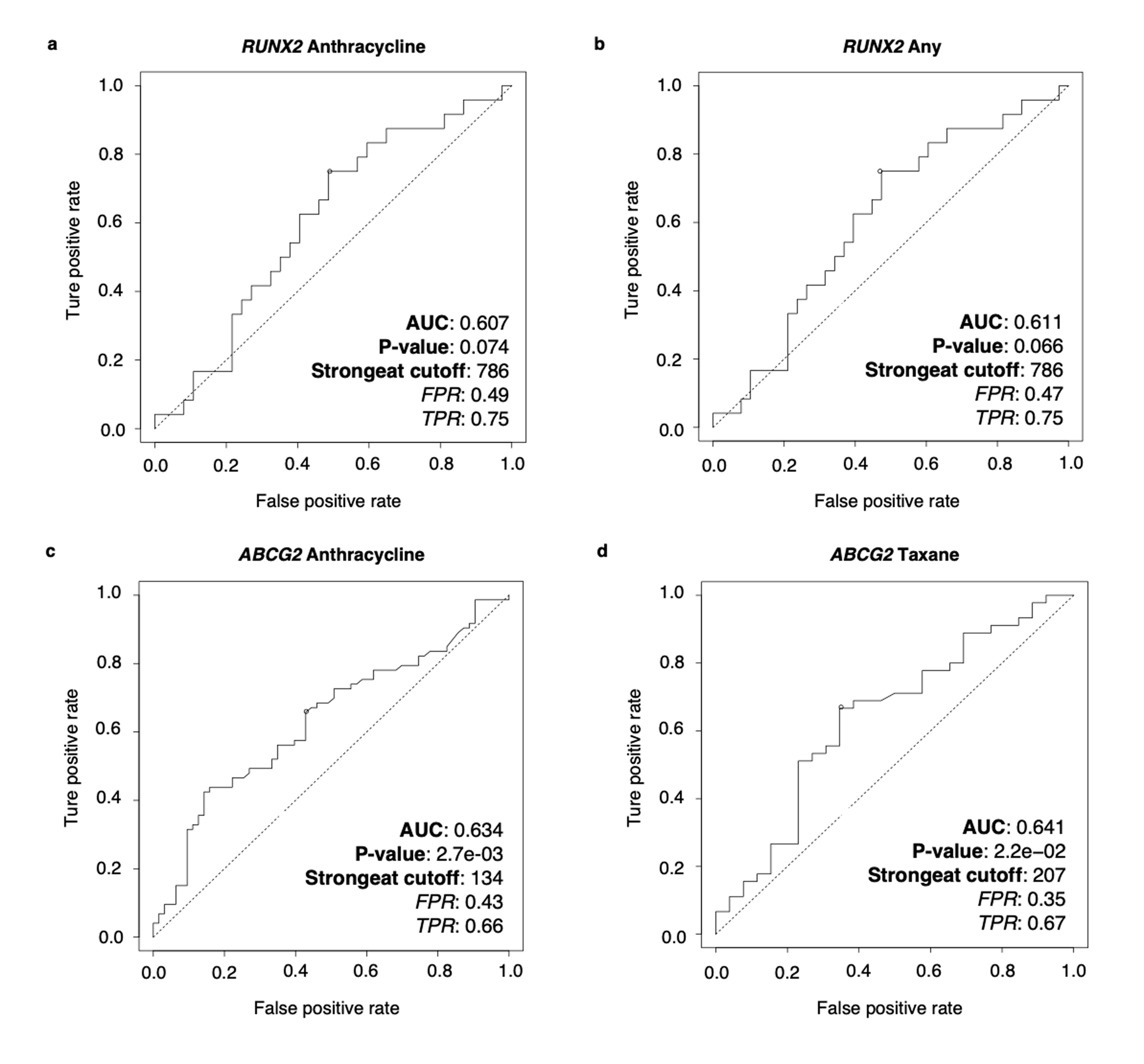

Supplement: Supplementary file 3 — ABCG2 and RUNX2 ROC plots for the indicated chemotherapy regime. (PNG 548 kb) [file 13402_2020_497_Fig8_ESM.png]

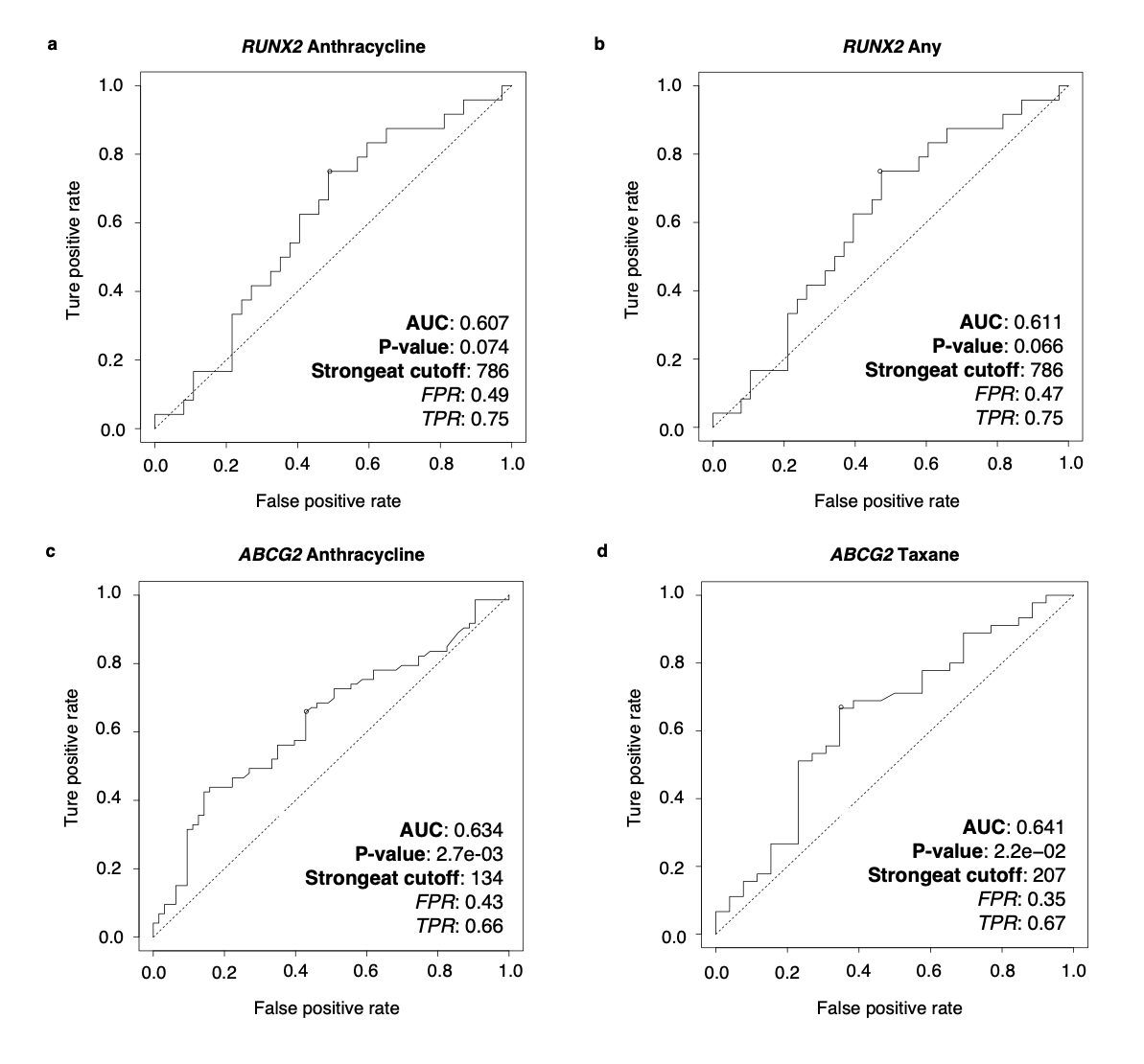

Supplement: Supplementary file 4 — High Resolution Image (TIFF 5173 kb) [file 13402_2020_497_MOESM2_ESM.tiff]

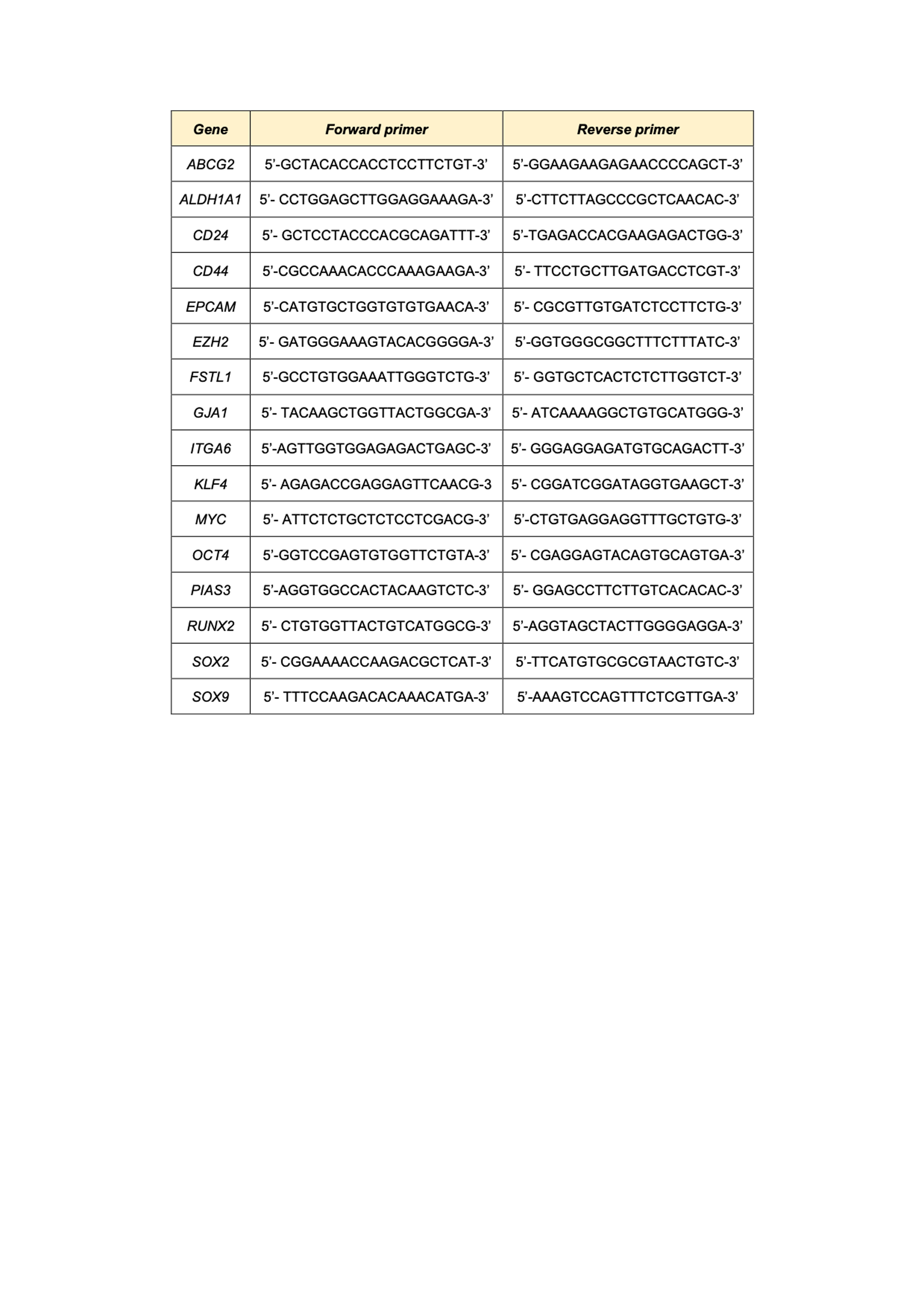

Supplement: Supplementary file 5 — Primer sequences. (PNG 481 kb) [file 13402_2020_497_Fig9_ESM.png]

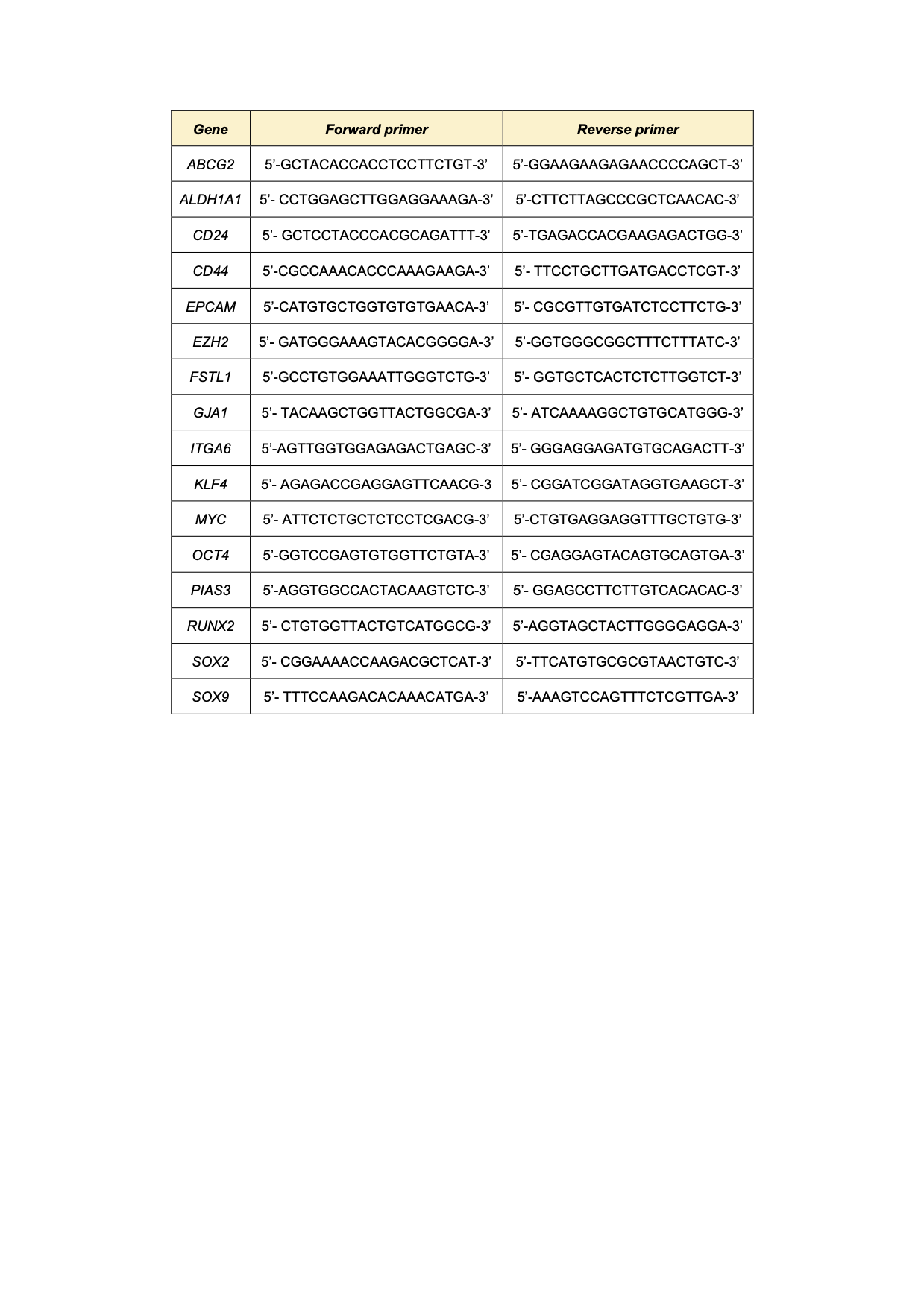

Supplement: Supplementary file 6 — High Resolution (TIFF 8490 kb) [file 13402_2020_497_MOESM3_ESM.tiff]

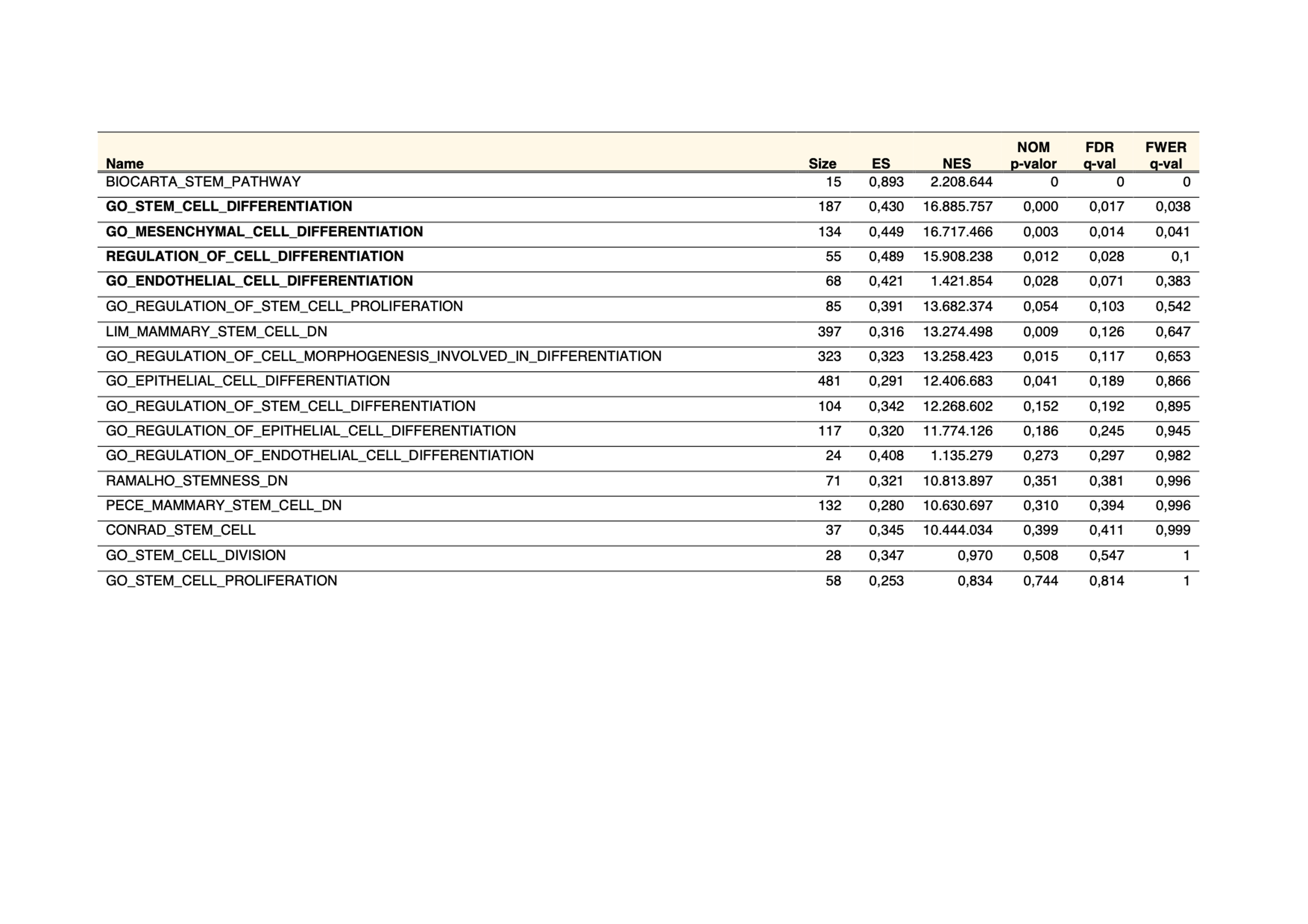

Supplement: Supplementary file 7 — Summary of the GeneSets analysis reports. Size: number of genes; ES: enrichment score; NES: normalized enrichment score; NOM p-valor: nominal p value; FDR q-val: false discovery rate; FWER q-val: familywise-error rate. (PNG 739 kb) [file 13402_2020_497_Fig10_ESM.png]

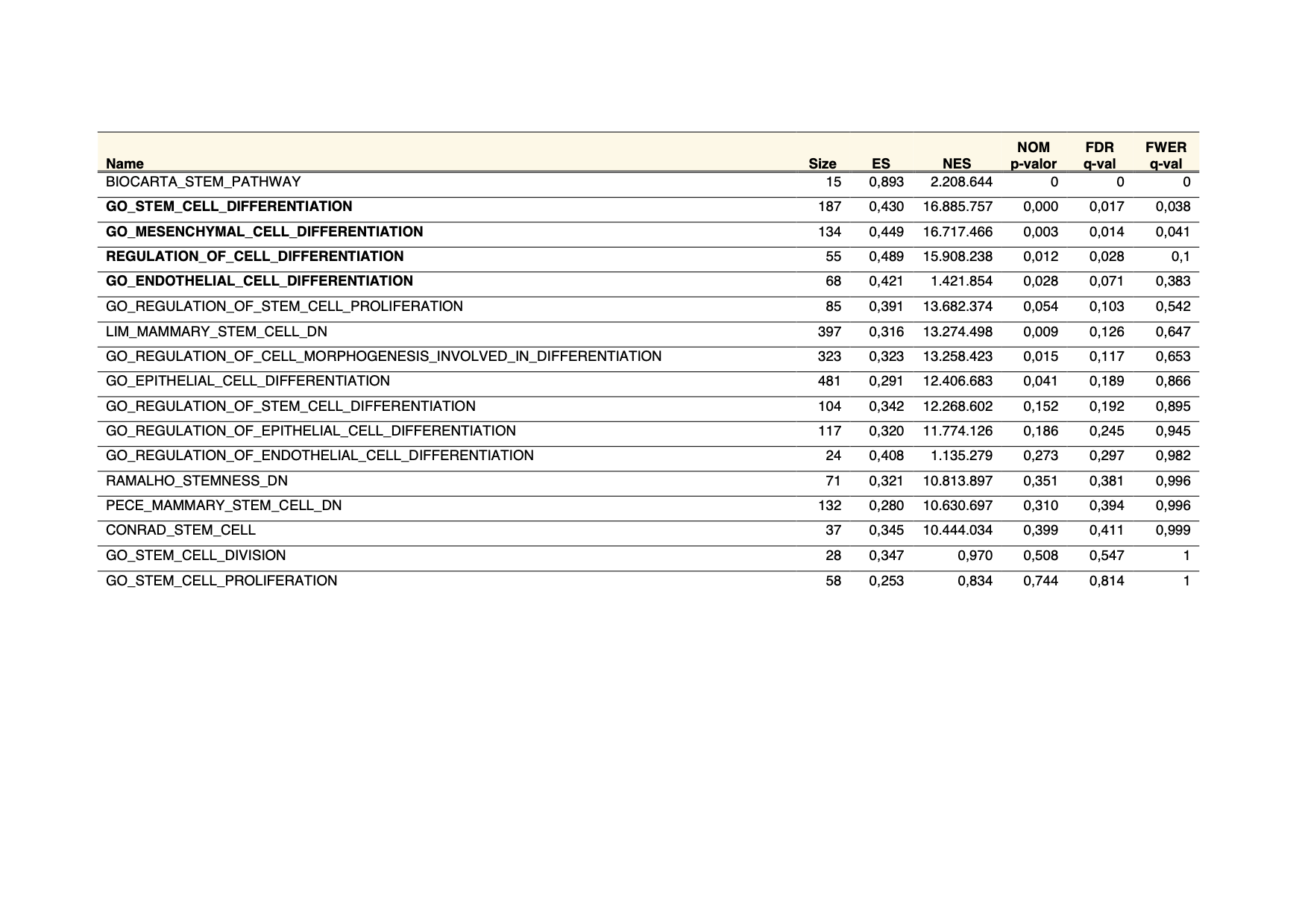

Supplement: Supplementary file 8 — High Resolution (TIFF 8490 kb) [file 13402_2020_497_MOESM4_ESM.tiff]

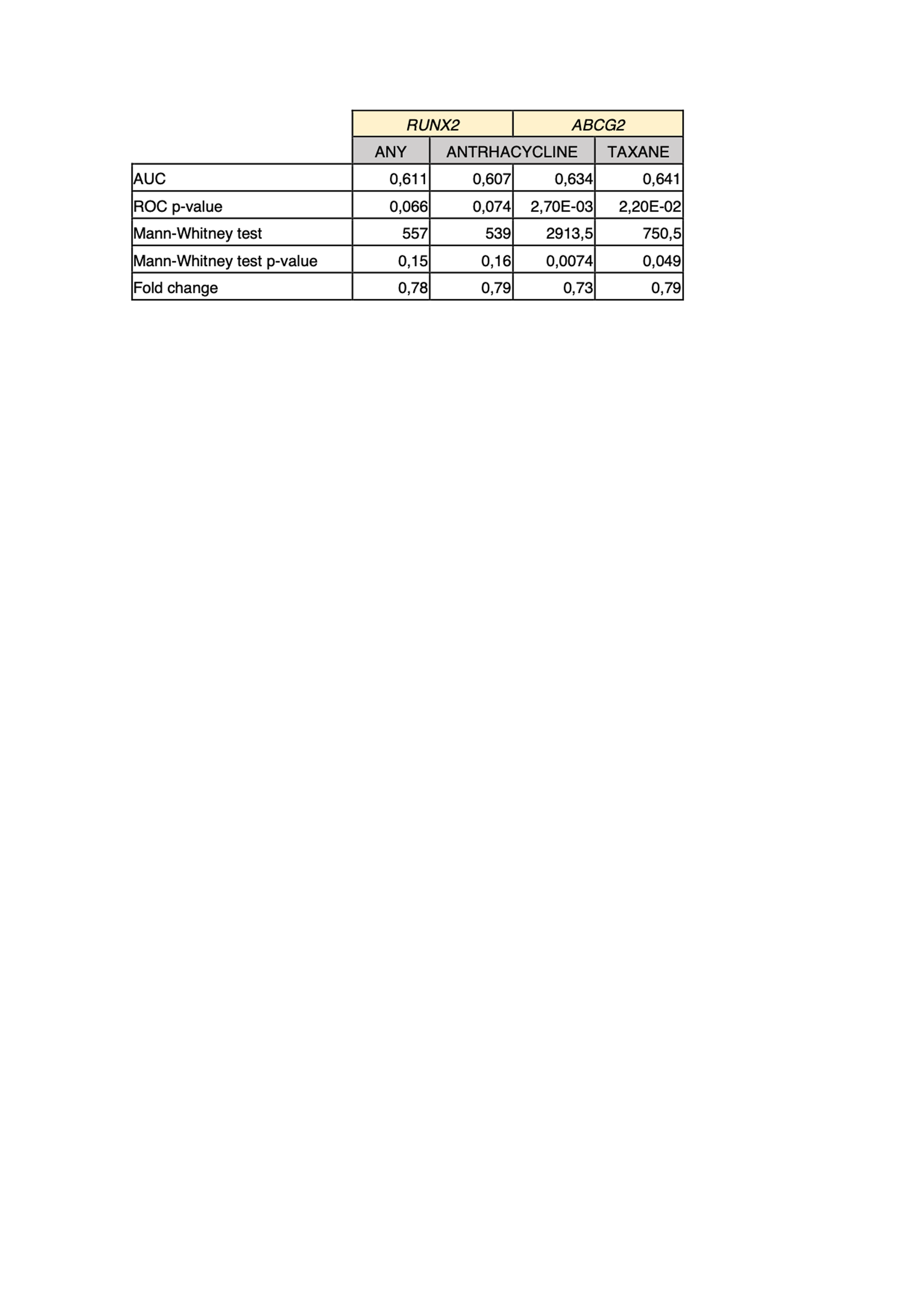

Supplement: Supplementary file 9 — Values of AUC, ROC p value, Mann-Whitney test, Mann- Whitney test p value and fold change indicating differences between responder (better RFS) and non-responder (worse RFS) patients based on RUNX2 and ABCG2 gene expression in basal-like breast cancer patients. (PNG 231 kb) [file 13402_2020_497_Fig11_ESM.png]

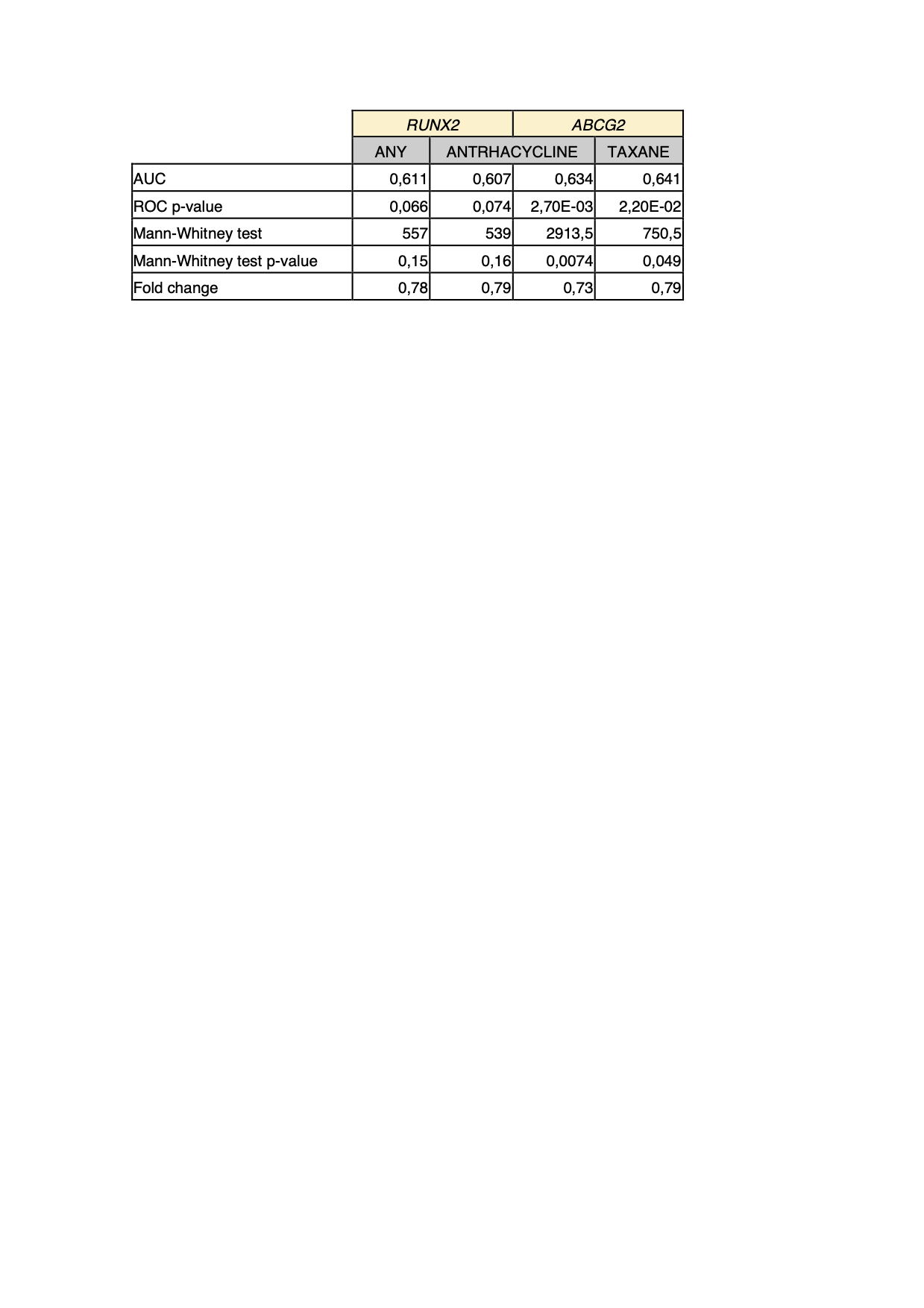

Supplement: Supplementary file 10 — High Resolution (TIFF 8490 kb) [file 13402_2020_497_MOESM5_ESM.tiff]
